# Modelling the evolution of genetic instability during tumour progression

**DOI:** 10.1111/eva.12024

**Published:** 2012-11-26

**Authors:** Ruchira S Datta, Alice Gutteridge, Charles Swanton, Carlo C Maley, Trevor A Graham

**Affiliations:** 1 Center for Evolution and Cancer, University of California San FranciscoSan Francisco, CA, USA; 2 Center of Mathematics and Physics in the Life Sciences and Experimental Biology (CoMPLEX), University College LondonLondon, UK; 3 Translation Cancer Therapeutics Laboratory, Cancer Research UK London Research InstituteLondon, UK

**Keywords:** carcinogenesis, clonal expansion, genetic instability, mathematical biology, selection

## Abstract

The role of genetic instability in driving carcinogenesis remains controversial. Genetic instability should accelerate carcinogenesis by increasing the rate of advantageous driver mutations; however, genetic instability can also potentially retard tumour growth by increasing the rate of deleterious mutation. As such, it is unclear whether genetically unstable clones would tend to be more selectively advantageous than their genetically stable counterparts within a growing tumour. Here, we show the circumstances where genetic instability evolves during tumour progression towards cancer. We employ a Wright–Fisher type model that describes the evolution of tumour subclones. Clones can acquire both advantageous and deleterious mutations, and mutator mutations that increase a cell's intrinsic mutation rate. Within the model, cancers evolve with a mutator phenotype when driver mutations bestow only moderate increases in fitness: very strong or weak selection for driver mutations suppresses the evolution of a mutator phenotype. Genetic instability occurs secondarily to selectively advantageous driver mutations. Deleterious mutations have relatively little effect on the evolution of genetic instability unless selection for additional driver mutations is very weak or if deleterious mutations are very common. Our model provides a framework for studying the evolution of genetic instability in tumour progression. Our analysis highlights the central role of selection in shaping patterns of mutation in carcinogenesis.

## Introduction

The role of genetic instability as a driver of tumorigenesis remains controversial. Recent whole-genome sequencing studies show that cancers frequently contain hundreds of putative *driver* mutations that are suggested to play a causative role in tumour development (Loeb 2011. Given the impressive replication fidelity of normal cells [estimated mutation rate of between 10^−9^ and 10^−10^ per base pair per division (Salk et al. 2010)], it has been argued that an elevated mutation rate is required to acquire this large number of carcinogenic mutations within a reasonable time (Loeb 2001). However, whilst this intuitive argument for a central role of genetic instability in tumorigenesis is appealing, it is likely to represent an oversimplification and as such may be misleading, for three key reasons. First, whilst an increase in the mutation rate accelerates the accumulation of tumour-causing mutations, it will also accelerate the accumulation of mutations deleterious for tumour growth (Cahill et al. 1999); therefore, an increase in the mutation rate could potentially cause retardation of tumour growth, a phenomenon termed *negative clonal selection* (Beckman and Loeb 2005). Indeed, breast cancers with the most extreme levels of chromosomal instability (CIN) have a better prognosis than cancers with more moderate levels of genetic instability (Birkbak et al. 2011; Roylance et al. 2011). Second, the development of a genetically unstable cancer, rather than a genetically stable cancer, requires that the tumour acquires additional (epi-)mutations in genome-destabilising genes (Tomlinson et al. 1996); if genetic instability is an initiating event, rate-limiting disruption to these instability genes must occur prior to rate-limiting driver gene mutations. Third, the argument neglects the effects of clonal expansion as a means to drive malignant progression; clonal expansion increases the number of cells susceptible to further mutations and so accelerates carcinogenesis (Tomlinson and Bodmer 1999; Luebeck and Moolgavkar 2002). Here, we investigate the evolution of an increased mutation rate during tumour progression using a mathematical model of tumour progression. We determine the circumstances that select for an increased mutation rate and the circumstances when an increased mutation rate accelerates tumour progression.

There is extensive and conflicting literature about the role of genetic instability in both the initiation of tumour growth (hereafter termed *initiation*) and in the progression of established tumours towards cancer (hereafter termed *progression*). Herein, we focus on the role of genetic instability in tumour progression. Marked genetic instability is certainly found in many cancer types: for example, 85% of colorectal cancers (CRCs) show CIN, and the remaining 15% have microsatellite instability (MSI) (Fearon 2011). Furthermore, chromosomally unstable CRCs typically have a worse prognosis than non-CIN tumours (Walther et al. 2008) and are more likely to be intrinsically multidrug resistant (Lee et al. 2011). Moreover, in the premalignant conditions Barrett's oesophagus and ulcerative colitis, aneuploidy is evident in nontumour tissue and is strongly associated with later tumour development (Rabinovitch et al. 1999; Maley et al. 2004). It is tempting to conclude a causative role for genetic instability in CRC progression from these data. However, it remains unclear whether or not CIN *per se* is selected in aggressive tumours, or if CIN tends to be associated with another highly selected phenotype, such as faster replication in the absence of error correcting, or some kind of intrinsic cellular hardiness, for example through a tolerance of abnormal gene dosing that is the consequence of aneuploidy. Interestingly, in evolving bacterial populations, sporadic evolving mutator phenotype strains can occasionally outcompete resident nonmutator strains (Sniegowski et al. 1997) producing a population of rapidly evolving cells (Barrick et al. 2009). Indeed, theoretical models show that mutator phenotypes can reach fixation in a population by ‘hitchhiking’ along with a selectively advantageous mutation (Taddei et al. 1997). Here, we examine the evolution of an increased mutation rate in a growing population of tumour cells, taking into account that additional mutations can be either advantageous or deleterious for the newly generated clone.

There are a number of theoretical models concerning the evolution of genetic instability in tumour progression. Beckman and Loeb's model suggests that carcinogenesis is more efficient with an elevated mutation rate, although the model does not consider the relative effects of clonal expansion of precancerous mutant clones (Beckman and Loeb 2006). Clonal expansion markedly alters the rate at which a tumour acquires driver mutations (Luebeck and Moolgavkar 2002; Hornsby et al. 2007), and so including this phenomenon is likely important. Furthermore, the prediction that an elevated mutation rate improves the *efficiency* of cancer production (Beckman 2010) (efficiency is defined in terms of the rate of carcinogenesis and incidence of cancer) suggests that a mutator phenotype can have a driving role in carcinogenesis, but does not address whether or not there is selection for a mutator phenotype *per se*. The Wright–Fisher type model of Beerenwinkel et al. described the acquisition of driver mutations that trigger clonal expansions within a tumour. They found that whilst an elevated mutation rate was not required for carcinogenesis to occur in a reasonable time, an increased mutation rate did reduce the waiting time to cancer (Beerenwinkel et al. 2007). A subsequent related analysis showed that selective advantage of driver mutations dominated the dynamics (Schollnberger et al. 2010). Beerenwinkel's model considered only advantageous mutations and had a fixed mutation rate. Beckman and Loeb's analysis of the effect of mutator phenotype-driven negative clonal selection in limiting tumorigenesis suggested negative selection was unlikely to be a limiting factor in the evolution of a mutator phenotype (Beckman and Loeb 2005); however, as this model did not simultaneously consider both advantageous and deleterious mutations, it remains unclear whether a mutator phenotype would be selected above a nonmutator phenotype when deleterious mutations can occur. Similarly, Beckman's model that described both deleterious and advantageous mutations in relation to a tumour cell's absolute fitness did not allow the mutation rate to naturally evolve (Beckman 2009). Komarova and Wodarz (2004) have investigated the role of a mutator phenotype in determining the efficiency of cancer growth that is initiated by a small number of mutations, whilst simultaneously considering deleterious mutation; they suggest that an initially high mutation rate that decreases later in progression increases the speed of carcinogenesis and make predictions as to the optimal mutation rate for carcinogenesis that balances deleterious versus advantageous mutation (Komarova et al. 2008). Komarova et al. models describe only the acquisition of an initial few driver mutations and do not describe intratumour clonal dynamics. Our model differs to these previous models by simultaneously considering both advantageous and deleterious mutations, whilst describing intratumour clonal dynamics and allowing the mutation rate to evolve.

Here, we address the role of genetic instability in driving tumour evolution using a model of clonal evolution within a growing tumour. Building upon previous modelling approaches, our model includes both ‘positive clonal selection’ that drives clonal expansions and ‘negative clonal selection’ that retards clonal expansion and permits the mutation rate to evolve during carcinogenesis. The model has a Wright–Fisher type construction, whereby the abundance of offspring from each intratumour clone is determined by the clone's relative fitness within the tumour. A cell's relative fitness is increased if it acquires additional driver mutations and is decreased by acquiring additional deleterious mutations. We use the model to consider how the strength of selection for driver mutations and against housekeeper mutations determines whether the genetic instability is required for efficient tumorigenesis. We also examine evidence for a selective pressure driving the evolution of an increased mutation rate in tumour progression, and so determine the circumstances and timing of switches to an elevated mutation rate.

## Methods

We developed a mathematical model describing the accumulation of advantageous, deleterious and neutral mutations by clones within a growing tumour. Our model is an extension of the model first proposed by Beerenwinkel et al. (2007) that described the accumulation of driver mutations by intratumour clones during tumour progression. Here, we have extended the Beerenwinkel model to describe multiple classes of mutations; both deleterious and advantageous mutations, and other mutation classes that do not directly impact on a cell's fitness. Following Beerenwinkel et al.*,* our model has a Wright–Fisher type construction, wherein the number of offspring of each clone is determined by the clone's relative fitness compared with all the other clones in the tumour. In this sense, the tumour is considered a ‘well-mixed’ population of cells, and spatial considerations, such as clonal interference (Martens et al. 2011), are ignored.

Fitness is determined by a cell's genotype: accumulating *driver* gene mutations increases a cell's fitness, whereas additional *housekeeper* gene mutations decrease a cell's fitness. Additionally, cells can acquire *mutator* gene mutations that have no direct effect on fitness, but cause the cell to have decreased DNA replication fidelity (i.e. a higher intrinsic mutation rate). Finally, cells can acquire selectively neutral *passenger* mutations that have no effect on fitness. Driver genes represent the proto-oncogenes and haploinsufficient tumour suppressor genes, housekeeper genes the set of genes that is needed for cell survival, such as those required to maintain ribosomes, and mutator genes the set of genes involved in DNA replication fidelity, such as the mismatch repair family of proteins. Passenger mutations should be considered as mutations in nonfunctional regions of the genome, such as in nongenic nonregulatory regions, within nonexpressed genes or nonfunctional intronic mutations.

Tumour growth begins with a population of 10^6^ cells. The simulation ends when a tumour reaches a maximum size of 10^9^ cells (which we would expect to have an approximate size of between 1 and 10 cm^3^) or when a cell has acquired *C* driver mutations. The size of the tumour cell population, *N*(*t*)*,* is constrained to grow exponentially such that:


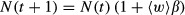
(1)

where *ß > 0* is a constant that sets the speed of exponential tumour growth, and <*w*> is the average fitness of the tumour population (defined below). To account for stochastic fluctuations in tumour size, in the simulations, the new size of the tumour at each timestep is sampled from a Poisson distribution with mean:


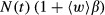


A tumour cell can acquire a maximum of *m*_*d*_ driver gene mutations, *m*_*h*_ housekeeper gene mutations, *m*_*p*_ passenger mutations and *m*_*m*_ mutator gene mutations. An additional driver gene mutation increases a cell's fitness by a factor of (1*+s*_*d*_), whereas an additional housekeeper gene mutation decreases fitness by a factor of (1*−s*_*h*_) and the effect of multiple mutations is multiplicative. Therefore, the relative fitness of a cell with *i* driver gene mutations, *j* housekeeper gene mutations, *k* passenger mutations and *l* mutator gene mutations is defined as:


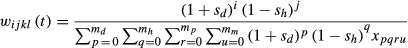
(2)

where 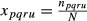 is the proportion of tumour cells that have *p*, *q*, *r* and *u* driver, housekeeper, passenger and mutator mutations, respectively.

The average fitness of the tumour population as a whole is then:


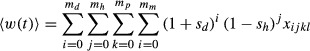
(3)

Mutator gene mutations act to increase a cell's intrinsic mutation rate. We consider just two states for the mutation rate; the baseline (low) rate of *μ*_*L*_ per gene per division, and an elevated rate *μ*_*H*_, that is, the consequence of mutator gene mutations. The switch from *μ*_*L*_ to *μ*_*H*_ occurs when a cell has acquired *M* mutator mutations.

The probability of sampling a cell with *i* driver gene mutations, *j* housekeeper gene mutations, *k* passenger mutations, irrespective of the number of mutator gene mutations is:


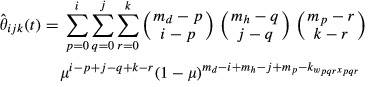


Consideration of the mutator genes leads to the probability of sampling a cell with *i*, *j*, *k*, *l* mutations in the respective classes as: If *l<M* then:


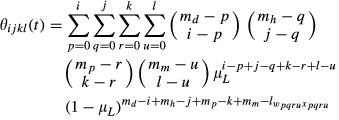
(4)

and if *l* ≥ *M* then:


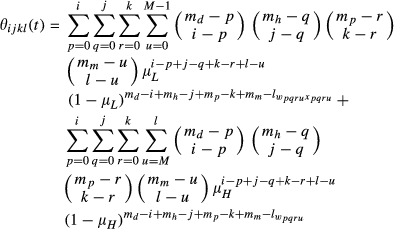
(5)

To simulate the model, the *θ*_*ijkl*_ are used to form a multinomial distribution describing the probability of sampling a mutant bearing *i*, *j*, *k* and *l* mutations in the respective classes in the next generation. The probability mass function is:


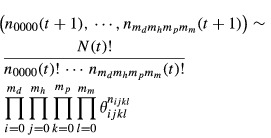
(6)

A sample from this distribution defines *n*_*ijkl*_ (*t* + 1).

For computational efficiency, a maximum of four mutations are permitted to occur in a single cell per division. To implement a maximum number of mutations per cell, the summations in eqns (4) and (5) begin at *p* =*i−*4, *q* = *j−*4, etc. The *θ*_*ijkl*_ are then normalised to sum to 1.

C code that simulates the model is available from the corresponding author on request.

## Results

**Figure 1:**
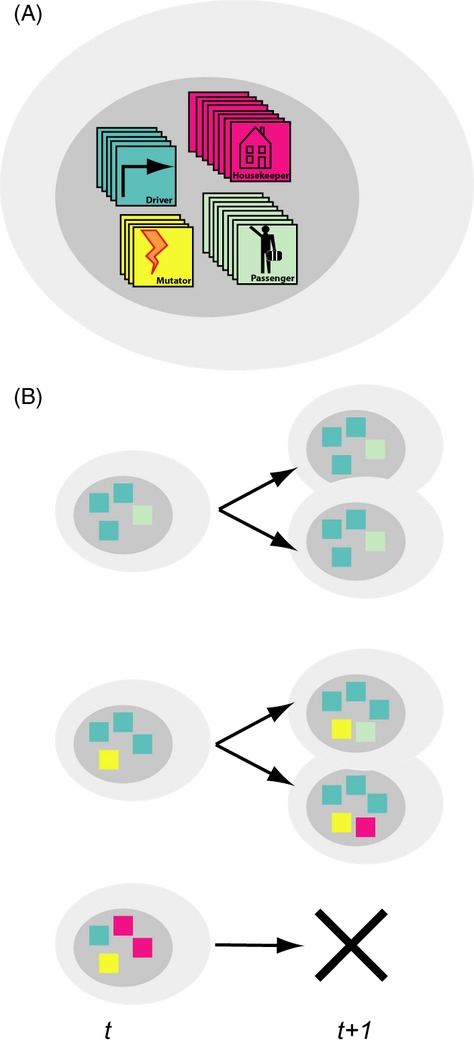
Cartoon of model construction. (A) Cells within the model can have driver mutations (depicted in blue) that, when mutated, cause an increase in the cell's fitness. Housekeeper gene mutations (depicted in pink) decrease a cell's fitness. Passenger mutations (green) have no effect on fitness, and the accumulation of mutator mutations (yellow) leads to an increase in the cell-intrinsic per-locus mutation rate. (B) Clonal evolution is modelled using a Wright–Fisher type construction, whereby the number of offspring of each cell in the next generation is a function of the cell's relative fitness within the tumour, which is in turn a function of the number of driver and housekeeper mutations borne by the cell. Cells with more driver mutations and fewer housekeeper mutations will tend to have more offspring in the next generation; too many housekeeper mutations will lead to clonal extinction.

A Wright–Fisher type model of intratumour clonal evolution was constructed. The model described the genetic progression of *tumour* cells, defined as non-invasive neoplastic cells, to *cancer* cells, defined as the cells capable of local invasion and metastasis. Within the model, cells could acquire selectively advantageous *driver* mutations, that increased the relative fitness of the cell by a factor of (1+*s*_*d*_), selectively disadvantageous *housekeeper* mutations that decreased fitness by a factor of (1−*s*_*h*_) and selectively neutral *passenger* mutations (Fig. 1). Cells could also acquire *mutator* mutations that led to an increase in the cell's mutation rate: after *M* mutator mutations occurred in a single cell, the cell's intrinsic per-locus mutation rate increased from *μ*_*L*_ to *μ*_*H*_ mutations per division. Mutations at each locus within a particular class (driver, housekeeper, passenger and mutator, respectively) were assumed to be equivalent, so it sufficed to track only the total number of mutations borne by each cell in each class of genes. A ‘clone’ was defined as the population of cells with a particular number (*i*, *j*, *k*, *l*) of mutations in the respective classes. The tumour population was assumed to grow exponentially. The number of offspring of each intratumour clone was a function of the clone's relative fitness within the tumour, as determined by the number of driver and housekeeper mutations borne by the clone; this gave the model a Wright–Fisher type construction. The tumour population was allowed to evolve under this model and the clonal structure and efficiency of tumorigenesis was examined under a range of model parameters.

**Figure 2:**
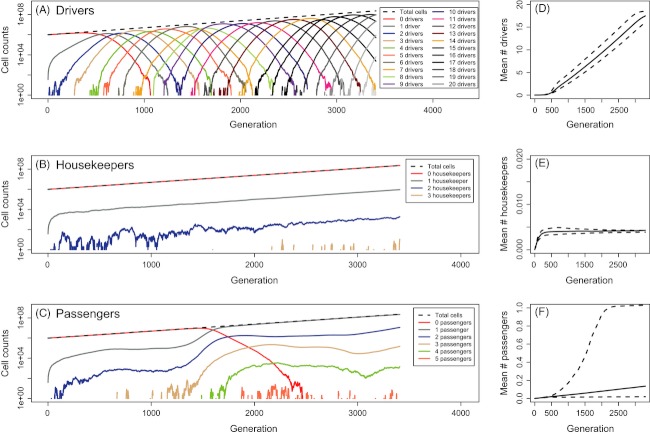
The pattern of mutation accumulation. (A) The sequential acquisition of driver mutations in a single run of the model. The acquisition of driver mutations shows a travelling wave pattern. Coloured lines show the abundance of clones with *i* = 0, 1, 2, … driver mutations, respectively, dashed line shows the total number of cells within the tumour. Model parameters: *m*_*d*_ = *m*_*h*_ = *m*_*p*_ = 100, *μ*_*L*_ = *μ*_*H*_ = 10^−7^, *s*_*h*_ = *s*_*d*_ = 0.01 and *β* = 0.016 (chosen to achieve exponential growth from 10^6^ to 10^9^ cells in ∼4500 generations as in Beerenwinkel et al.). (B) Acquisition of housekeeper mutations in the parameter regime described in A. Housekeepers do not accrue to a significant level: the bulk of the tumour is composed of a clone with no housekeeper mutations (red line). (C) Acquisition of passenger mutations in the parameter regime described in A. The plummeting red line shows a clone with no passenger mutations being driven to extinction. A clone marked with a single passenger mutation (grey line) has an acquired additional driver mutation and so clonally expands to dominance. (D) Average (mean) number of driver mutations in a tumour as a function of time. Driver mutations accrue continuously with time. Mean values were calculated as an average across 10 000 runs with parameters described in (A); dashed lines represent the 95% quantiles of mean simulation values of those 10 000 runs. (E) Average number of housekeeper mutations in a tumour as a function of time. Housekeeper mutations do not accrue in significant numbers nor produce clones of large sizes. Dashed lines are 95% quantiles as described in (D). (F) Average number of passenger mutations in a tumour as a function of time. Passenger mutations accrue linearly with time. Dashed lines are 95% quantiles as described in (D).

The model exhibited ‘travelling waves’ of the growth and then subsequent extinction of clones bearing increasing numbers of driver mutations (Fig. 2A,D), as had been previously reported by Beerenwinkel et al. (2007). The wave patterns were due to clonal competition; each new driver mutation triggered clonal expansion and drove the previously resident cells to extinction. Deleterious mutations did not accrue; cells with a large number of driver mutations could tolerate only a few deleterious (housekeeper) mutations without being outcompeted in the tumour by clones without deleterious mutations (Fig. 2B,E). Passenger mutations accrued in a linear fashion with respect to time but typically remained at low numbers in the tumour (Fig. 2C,F). Stochastic fluctuations were observed in passenger mutation numbers (Fig. 2C), as clonal expansion of passengers was caused by their hitchhiking along with a clone that had acquired a new driver mutation.

### An increased mutation rate markedly accelerates tumorigenesis only when selection for driver mutations is weak

The effect of an increased mutation rate on the rate of cancer evolution was examined. Mutator mutations were not considered here, so that the mutation rate remained fixed throughout the simulation; hereafter, the model with a fixed mutation rate is referred to as ‘model 1’. A cancer was defined as having grown when at least 10% of the tumour was composed of cells with at least 20 driver mutations. The number of driver mutations is likely to differ significantly between tumours; the requirement for 20 driver mutations represented the estimated maximal driver mutation burden in colorectal cancer (Sjöblom et al. 2006) and is in accordance with previous models (Beerenwinkel et al. 2007). Runs of the model were terminated when the tumour population exceeded 10^9^ cells; the probability of developing cancer was defined as the proportion of runs (with a particular set of parameters) that led to the development of cancer before the tumour as a whole was composed of more than 10^9^ cells.

**Figure 3:**
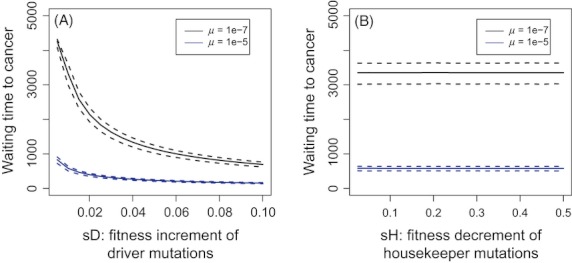
The effect of an increased mutation rate as a function of selection. (A) Waiting time to cancer for *μ* = 10^−7^ compared with *μ* = 10^−5^ for a range of driver mutation advantages (values of *s*_*d*_). The deleterious effect of housekeeper mutations was set at *s*_*h*_ = 0.01 for all values of *s*_*d*_. The waiting time to cancer was reduced by an elevated mutation rate. The mutation rate has the greatest effect on the waiting time to cancer when selection for new driver mutations was weak. Increased selection for new driver mutations caused a more pronounced reduction in the waiting time to cancer when the mutation rate was lower. Simulations for *m*_*d*_ = *m*_*h*_ = *m*_*p*_ = 100, averaged over 10 000 runs. (B) Waiting time to cancer for a variety of housekeeper selective disadvantages. When the number of driver and housekeeper loci was equal, deleterious housekeeper mutations had minimal effect on the waiting time to cancer, and the effect was independent of the strength of deleterious selection.

Carcinogenesis was faster (Fig. 3A) when the basal mutation rate was higher. However, the relative increase in efficiency of tumorigenesis due to an elevated mutation rate was reduced by strong selection for additional driver mutations (Fig. 3A). In other words, when additional driver mutations caused a strong selective advantage, a mutator phenotype was not necessary for efficient tumorigenesis. Relatedly, an increased selective advantage for driver mutations caused a bigger reduction in the waiting time to cancer when the mutation rate was lower.

The simulations here were performed with equal numbers of housekeeper and driver mutations. In this case, deleterious housekeeper mutations had little effect on the rate or likelihood of carcinogenesis (Fig. 3B). Cells acquiring a deleterious housekeeper mutation were rapidly outcompeted and driven to extinction by more fit clones. As the probability of a cell acquiring a deleterious mutation was comparable to that of the cell acquiring an advantageous mutation, negative clonal selection impeded the growth of only rare cells within the most aggressive clone, and so failed to impede tumour growth. This result highlights that clonal selection was the dominant force within the model.

### The selective advantage of driver mutations determines whether a mutator phenotype evolves

The evolution of a mutator phenotype within a growing cancer was examined. The model where the mutation rate could vary is referred to as model 2. The switch to a mutator phenotype, whereby a cell's intrinsic mutation rate increased from *μ*_*L*_ = 10^−7^ to *μ*_*H*_ = 10^−5^ per-locus per division, occurred when a cell had acquired at least *M* mutator mutations. The model was simulated for a range of values of *M*, and for a range of values of *s*_*d*_, the fitness increment bestowed by an additional driver mutation. For these simulations, the number of driver and housekeeper loci was set to *m*_*d*_ = *m*_*h*_ = 100.

**Figure 4:**
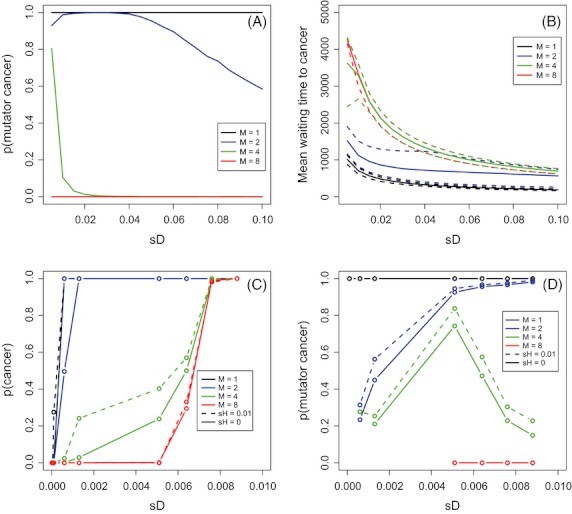
Selection and the evolution of an increased mutation rate. (A) Probability that cancer will have evolved a mutator phenotype as a function of the relative fitness benefits of additional driver mutations. Strong selection for additional driver mutations suppressed the evolution of a mutator phenotype, and this effect was exaggerated when multiple mutator gene mutations are required to develop genetic instability. *s*_*h*_ = 0.01, *m*_*d*_ = *m*_*h*_ = *m*_*p*_ = 100, *μ*_*L*_ = 10^−7^ and *μ*_*H*_ = 10^−5^. Simulations averaged over 10 000 runs. (B) Mean waiting time to cancer as a function of the relative fitness benefits of additional driver mutations (averaged over 10 000 runs of the model). Tumorigenesis tended to be faster when a mutator phenotype could readily evolve, or when selection for driver mutations was strong. Dashed lines represent 95% quantiles of the simulated values. (C) Probability of cancer for very weak selective advantages of driver mutations. Simulations averaged over 750 runs. Cancer was more likely when a mutator phenotype could evolve. Selection against housekeeper mutations reduced the incidence of cancer (solid lines: *s*_*h*_ = 0.01; dashed lines: *s*_*h*_ = 0). (D) Probability that a cancer will have evolved a mutator phenotype as a function of selection. For very weak or very strong driver gene selective advantage, a mutator phenotype is less likely to evolve.

Cancers were more likely to have evolved a mutator phenotype when selection for additional driver mutations was moderate *s*_*d*_∼0.01; strong selection for additional driver mutations suppressed the evolution of a mutator phenotype (Fig. 4A). Requiring more mutator mutations to develop a mutator phenotype (increasing *M*) prevented the evolution of genetic instability as cells had to acquire more intrinsically selectively neutral mutator mutations through genetic drift (Fig. 4A). Notably, with *M* = 1 cancers always had a mutator phenotype, whereas with *M* = 8 cancers never had a mutator phenotype. Being able to develop a mutator phenotype accelerated tumorigenesis (Fig. 4B), although the accelerating effect was reduced when selection for additional driver mutations was strong. For *s*_*d*_ ≥ 0.01, deleterious selection against housekeeper mutations had negligible impact on the likelihood of evolving a cancer with a mutator phenotype or on the waiting time to cancer (Fig. S1).

Requiring fewer mutations for cancer development reduced the waiting time to cancer (Fig. S2A) as fewer driver mutations needed to accrue. Correspondingly, the likelihood of the cancer having evolved a mutator phenotype decreased when fewer driver mutations were required for cancer (Fig. S2B). This was because the likelihood of a mutator clone hitchhiking to clonal dominance on a selective sweep of a driver mutation was reduced when there were fewer selective sweeps.

When selection for additional driver mutations was very weak (regions where *s*_*d*_ < 0.01; whereby an additional driver mutation had less than a 1% effect on the probability of the cell producing surviving offspring), the role of the mutator phenotype in carcinogenesis was altered. In this case, deleterious mutations reduced the likelihood of carcinogenesis (Fig. 4C); in regimes of very weak selection, clonal expansion was slow and so housekeeper mutations could significantly impede clone growth. Indeed, cancers were more likely to have evolved a mutator phenotype when *s*_*h*_ = 0 than when *s*_*h*_ = 0.01 (Fig. 4D). This was a consequence of the rate at which deleterious mutations accrued in these two parameter regimes: the increased probability of acquiring deleterious mutations appeared to reduce the advantage of the mutator phenotype when *s*_*h*_ > 0. As the selective benefit of an additional driver mutation was increased from very low *s*_*d*_ to high *s*_*d*_, the proportion of cancers evolving a mutator phenotype increased to a peak value and then decreased (Fig. 4A,D), reflecting the likelihood of sufficient mutator mutations accruing by drift in the most aggressive clone; with *M* = 1 cancers always evolved a mutator phenotype.

**Figure 5:**
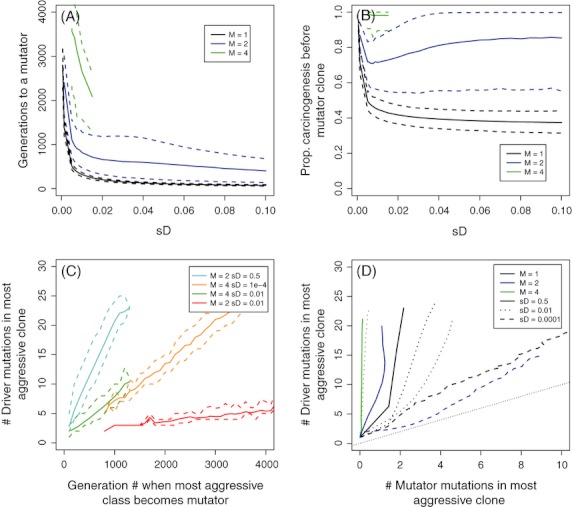
Timing of mutator phenotype evolution. (A) Generation when a mutator clone is established in the tumour (composes more than 10% of the tumour), as function of the strength of selection for driver mutations, for different numbers of mutator mutations required for a mutator phenotype. Requiring more mutator mutations to switch to a mutator phenotype increased the generations elapsed before a mutator clone is established. Stronger selection for driver mutations made a mutator clone evolve in fewer generations. Solid lines are simulation means, dashed lines represent 95% quantiles of the simulations. (B) Proportion of the time taken for a cancer to evolve before a mutator clone is established. Requiring more mutator mutations for a mutator phenotype caused the mutator phenotype to develop later in progression. For moderate to strong selection, increasing the strength of selection for driver mutations caused only marginal change in the relative timing of a mutator clone. Solid lines are simulation means, dashed lines represent 95% quantiles of the simulations. (C) Number of driver mutations in the most aggressive clone (clone with most driver mutations) at the time that it acquires a mutator phenotype. Clones acquired significant numbers of driver mutations before switching to a mutator phenotype. Dashed lines are 95% quantiles of the simulations. (D) The accumulation of driver mutations versus mutator mutations in the most aggressive clone. Clones acquired driver mutations at a much faster rate than mutator mutations. When selection for driver mutations was weak, driver mutations accrued at a more comparable rate to mutator mutations, indicating that genetic drift became a stronger influence in the clonal dynamics. Solid lines represent strong selection for additional driver mutations (*s*_*d*_ = 0.05); dotted lines moderate selection (*s*_*d*_ = 0.01); dashed lines weak selection (*s*_*d*_ = 10^−4^). *m*_*d*_ = *m*_*h*_ = *m*_*p*_ = 100, *μ*_*L*_ = 10^−7^, *μ*_*H*_ = 10^−5^ for all simulations. Data reported from aggregate measures of at least 100 runs.

The development of a mutator phenotype was frequently not an early event in tumour progression. Requiring a greater number of mutator mutations to switch to a mutator phenotype delayed the establishment of a clone with a mutator phenotype, both in absolute numbers of generations (Fig. 5A), and in terms of the proportion of the time taken for carcinogenesis that had elapsed before the mutator clone appeared (Fig. 5B). The strength of selection had a less-marked effect on the time at which a mutator clone formed. The clone with the most driver mutations (termed the most aggressive clone) accrued a significant number of driver mutations prior to developing a mutator phenotype; when additional driver mutations caused large increases in fitness, the number of driver mutations accrued prior to a mutator phenotype increased (Fig. 5C). Indeed, driver mutations always accrued more quickly than mutator mutations, and the difference in rate was increased as the selective benefit of additional driver mutations increased (Fig. 5D). These data reflect that mutator mutations were selectively neutral, and consequently that mutator clones formed only after mutator mutations had hitchhiked to prominence on the back of clonal expansions driven by the selected driver mutations.

### Abundant deleterious mutations slow carcinogenesis

**Figure 6:**
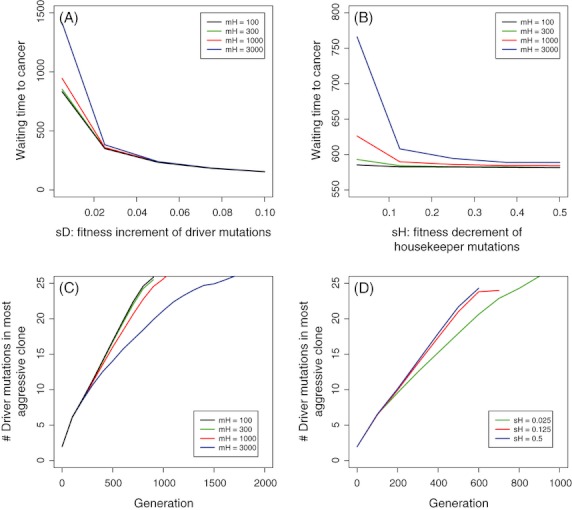
Abundant housekeeper mutations. (A) Mean waiting time to cancer as a function of the relative selective advantage of driver mutations. Abundant deleterious housekeeper loci increased the waiting time to cancer. When selection for additional driver mutations was strong, additional housekeeper loci had only a very slight effect on the waiting time to cancer. (B) Mean waiting time to cancer as a function of the relative selective disadvantage of housekeeper mutations. Stronger selection against housekeeper mutations slightly accelerated carcinogenesis. (C) Accrual over time of the number of driver mutations in the most aggressive clone for different numbers of housekeeper loci. Driver mutations accrued more slowly as the abundance of housekeeper mutations increased. Data from simulations with *s*_*d*_ = 0.005, *s*_*h*_ = 0.01. (D) Accrual over time of the number of driver mutations in the most aggressive clone for different values of the strength of selection against housekeeper mutations. Stronger selection against housekeeper mutations increased the rate at which driver mutations accrued. Data from simulations with *s*_*d*_ = 0.01, *m*_*h*_ = 3000. Model 1 (fixed mutation rate) was used to produce these data. For all runs, *m*_*d*_ = *m*_*p*_ = *m*_*m*_ = 100, *μ* = 10^−5^ for all simulations. *s*_*h*_ = *s*_*d*_ = 0.01 unless otherwise stated. Data reported from aggregate measures of 10 000 runs.

To look more closely at the impact of deleterious housekeeper mutations on carcinogenesis (negative clonal selection), simulations were performed with large numbers of housekeeper loci (*m*_*d*_ ≪ *m*_*h*_). This represented the situation where most mutations where likely to be deleterious, and where driver mutations were relatively rare. In model 1 (fixed mutation rate), increasing the number of housekeeper loci increased the waiting time to cancer (Fig. 6A,B). The longer waiting time for cancer due to increasing numbers of housekeeper genes was reduced as the selective advantage of driver mutations increased (Fig. 6A) as deleterious mutations impeded clone growth, and so in turn reduced the effective rate at which driver mutations accrued (Fig. 6C).

Interestingly, for a given number of housekeeper genes, increasing the strength of deleterious selection against housekeeper mutations led to a slight decrease in the waiting time to cancer (Fig. 6B). When deleterious mutations were particularly lethal for a cell, the fitness differential between deleteriously mutated and nondeleteriously mutated cells was marked. As such, cells that avoided deleterious mutations were relatively very fit within the tumour where deleterious mutations were abundant – the nondeleteriously mutated clones effectively received a fitness boost – and so grew to the detriment of deleteriously mutated clones. Thus, driver mutations accrued faster as the deleteriousness of housekeeper mutations was increased (Fig. 6D).

Using model 2, the effect of abundant deleterious mutations on the evolution of an increased mutation rate was examined. In this case, increasing the abundance of housekeeper loci decreased the likelihood that the tumour would evolve a mutator phenotype (Fig. S3). The strength of selection for new driver mutations determined whether a mutator phenotype would evolve; very strong or very weak selection for driver mutations suppressed the evolution of a mutator phenotype (Fig. S3A). Strong negative clonal selection against deleterious housekeeper mutations suppressed the evolution of a mutator phenotype (Fig. S3B).

### The evolution of genetic diversity

Genetic diversity within the tumour was quantified using three ecological measures: the Shannon index (a measure of the uncertainty in predicting the genotype of a sampled cell), the Simpson index (approximates the probability of drawing two cells that have the same genotype; smaller values indicate a more diverse population) and the richness (the number of different clones). Model 1 (fixed mutation rate) was used for the simulations of genetic diversity, with the mutation rate *μ* = 10^−5^ or *μ* = 10^−7^ and various values of the selective advantage of driver mutations *s*_*d*_.

**Figure 7:**
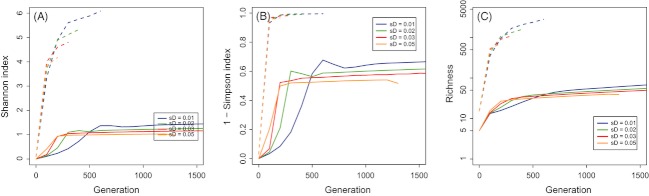
The evolution of genetic diversity. (A–C) Shannon, Simpson and richness indexes as function of time as the initiation of tumour growth for a fixed mutation. Diversity initially increases over time until reaching a plateau where the level of diversity is increases only slowly over time. Strong selection for additional driver mutations leads, eventually, to a less diverse tumour population. A higher mutation rate causes more diversity. 1-Simpson index is plotted to better illustrate the increase in diversity over time. Solid lines: *μ* = 10^−7^, dashed lines *μ* = 10^−5^. Simulations averaged over 10 000 runs.

For all parameter values, diversity initially increased sharply before reaching a pseudo -plateau where diversity increased only slowly over time (Fig. 7). The pseudo-plateau level of diversity was defined both by the fitness gain of additional driver mutations *s*_*d*_ and by the basal mutation rate *μ*. Strong selection for additional driver mutations reduced the pseudo-plateau levels of genetic diversity, whilst higher mutation rates increased the pseudo-plateau level of diversity. Strong selection caused clonal expansions to happen faster, causing the tumour to more rapidly become homogeneous for the new mutation and so suppressing diversity. A higher mutation rate led to the more rapid generation of new, genetically diverse, mutants. The mutation rate had a marked effect on the clonal diversity of a tumour; for instance, when measured by the Shannon index, the diversity measure at *μ* = 10^−5^ was approximately sixfold higher than the diversity at *μ* = 10^−7^. In comparison, a fivefold increase in the selective benefit of a new driver mutation caused about a 1.5-fold decrease in the final diversity of the tumour. In the initial phase of tumour growth, diversity increased more quickly for higher values of *s*_*d*_, likely reflecting the more rapid subclone growth for these parameter regimes.

## Discussion

Here, we have examined the evolution of a mutator phenotype during tumour progression from a benign lesion to a cancer. We have determined circumstances when, a mutator phenotype evolves during carcinogenesis, and looked at the effect of an evolving mutator phenotype on the efficiency of tumorigenesis.

Our model predicts that the strength of selection for driver mutations determines whether a cancer is likely to evolve a mutator phenotype: in situations where selection for driver mutations is very weak, or very strong, a mutator phenotype is unlikely to evolve. Previous studies have noted the central role of selection, predominating over the mutation rate, in driving the speed of carcinogenesis (Schollnberger et al. 2010). Indeed, an agent-based modelling study found that when both neutral and driver mutations were necessary for cancer formation, requiring more driver mutations to form a cancer actually increased the probability of cancer formation, as the rapid clonal expansions induced by the extra driver mutations made it more likely that a neutral mutation would be able to hitchhike to dominance within the neoplasm (Maley and Forrest 2000). Our study illustrates how the fitness advantage of driver mutations determines whether a mutator phenotype is likely to evolve at all. In the case of strong selection for additional driver mutations, the growth of the most aggressive clone (the clone with the most driver mutations) is very rapid, so that many cells that are ‘susceptible’ to acquiring the next driver mutation are rapidly produced. Mutator mutations are assumed to be selectively neutral in the model; mutator mutations therefore accrue only through genetic drift or by hitchhiking on the most aggressive clones. When two or more mutator mutations are required to generate the mutator phenotype [as would be the case for recessive mismatch repair (MMR) genes (Markowitz and Bertagnolli 2009)], the rate at which mutator genes drift to dominance within the most aggressive clone is much slower than the rate at which the most aggressive clone acquires the next driver mutation. In this way, mutator mutations are effectively removed from the tumour population as a result of their being outcompeted by the most aggressive clones. When selection for additional driver mutations is moderate, cells carrying mutator gene mutations are not outcompeted so rapidly, and so a mutator phenotype can evolve. A mutator cell then acquires driver mutations faster than its nonmutator counterparts, and so the mutator phenotype indirectly bestows a selective advantage. When selection for additional driver mutations is very weak, intratumour clone dynamics are driven nearly entirely by drift (not clonal selection). In this case, acquiring a mutator phenotype, which still accelerates the rate of driver gene mutation accumulation, does little to accelerate the rate of clone growth, and so selection for mutator cells is weak.

In the light of this result, it is instructive to consider what order of fitness advantage is bestowed by driver mutations within tumours. There is a lack of empirical measurements of the relative fitness of different tumour clones, although modelling efforts have suggested that drivers may cause only slight increases in fitness. Beerenwinkel et al. (2007), using their mathematical model of clonal evolution, suggested a relative fitness advantage of about 1% per driver mutation (i.e. *s*_*d*_ = 0.01; making cells 1% more likely to have surviving offspring) was sufficient to cause progression of colorectal adenomas to cancers in a reasonable time. Similarly, in their study of subclone expansion, Siegmund et al. (2011) saw only evidence for weak selection in CRCs. Bozic et al. (2010) estimated a fitness advantage of only about 0.4% per driver mutation for glioblastoma and pancreatic cancers. These predictions are in the ‘moderate’ range of selective advantages in our model. Furthermore, given the disparity in the number of putative driver mutations found in different types of cancer (Salk et al. 2010), it is likely that the fitness advantage of driver mutations differs significantly between cancer types. Thus, whether or not genetic instability is a hallmark of a particular cancer type may reflect, to some degree, the strength of selection for new driver mutations in the cancer type. Of course, fitness advantages will also differ between mutations at different loci, even within the same cancer type.

We have assumed that all driver mutations have the same consequence for a cell's fitness. In reality, mutations of different genes will have different consequences for fitness; for example, that distinct mutations of the *SETD2* gene are found in different intratumour clones in the same renal carcinoma (Gerlinger et al. 2012) indicates a strong selective pressure for inactivation of this particular gene. Consequently, the rate of clonal evolution within an individual tumour may change over time, and so the likelihood of evolving a mutator phenotype may change over time. For example, it is likely that the driver mutations with the largest selective effects would expand initially (as they would outcompete the clones with weaker driver mutations), leading to weaker driver mutations later in progression. In this case, genetic instability would be unlikely to evolve at the onset of tumour growth and would likely feature in the later stages of carcinogenesis. Indeed, this appears to be the case in Barrett's oesophagus where the detection of aneuploidy is a strong predictor of cancer development risk (Maley et al. 2004, 2006).

Unless deleterious mutations were very common, they did little to impede the progression of the most aggressive clones, supporting Beckman and Loeb's previous conclusion (Beckman and Loeb 2005). In fact, we observed that carcinogenesis could be actually *accelerated* by increasing the strength of selection against deleterious mutations, as strong selection against deleterious mutations served to increase the relative fitness of clones without deleterious mutations. This observation highlights how competition within tumours likely influences the pattern of clonal evolution. We had initially hypothesised that the rapid accrual of deleterious mutations would inhibit the growth of mutator clones, as have others previously (Cahill et al. 1999; Tomlinson and Bodmer 1999; Komarova and Wodarz 2004; Komarova et al. 2008). Whilst this was the case when selection for additional driver mutations was very weak, or if deleterious mutations were very common, moderate to strong selection for driver mutations caused cells that had acquired deleterious mutations to be rapidly outcompeted and removed from the tumour, particularly in the case where driver and housekeeper loci were of equal abundance. Increasing the likelihood of a cell acquiring a deleterious mutation (by increasing the abundance of housekeeper loci in each cell) did slow tumorigenesis, and indeed suppress the evolution of a mutator phenotype, indicating that deleterious mutations can act as a brake on carcinogenesis. However, the strength of selection for driver mutations dominated the dynamics of the time to cancer and the emergence of a mutator phenotype. Our model predicts that deleterious mutations will not accrue within tumours. This hypothesis could be tested by examining ‘cancer genomes’ for evidence of clonal mutations (high frequency within the tumour) that are predicted to impede cell growth or survival, or genetically engineering them into cells.

Tumours that developed a mutator phenotype, when more than one mutator mutation was required to switch to a mutator phenotype, tended to develop it after the tumour had acquired a significant driver mutation burden, which was typically late in tumour progression. This time-delay was due to the time taken for mutator mutations to accrue by drift, or by hitchhiking, in the most aggressive subclones. Therefore, our model predicts that if tumour growth is initiated without an increased mutation rate (Bodmer et al. 2008), then a mutator phenotype is likely to feature only later in progression, which is again consistent with the data from Barrett's oesophagus (Maley et al. 2004, 2006).

When only a single mutator mutation is required to switch to a mutator phenotype (case *M* = 1), cancers always evolved with a mutator phenotype and carcinogenesis was universally faster. Thus, our model predicts that mutator phenotypes drive efficient carcinogenesis and that mutator mutations should be selected in tumours. This supports the conclusion of Beckman and Loeb (Beckman and Loeb 2006). The case *M* = 1 represents the scenario when mutator genes have a dominant effect; that is, mutating a single copy of the mutator gene causes a switch to a mutator phenotype. However, whether or not mutator genes act dominantly in cancers is uncertain. The mismatch repair genes operate in a recessive fashion (Markowitz and Bertagnolli 2009) and the genetic basis of other mutator phenotypes seen in cancer is uncertain. It is noteworthy that in model systems, there are examples of dominant negative mutations in *TP53* that are associated with genetic instability (Song et al. 2007).

The level of genetic diversity within a tumour was related to both the selective advantage attributable to driver mutations and the basal mutation rate. Thus, our model predicts that genetic diversity within a tumour is a proxy measure of both the mutation rate and strength of clonal selection. Similarly, the model of Durrett et al. (2011) predicted that the degree of genetic diversity within a tumour was largely determined by the age of the tumour, and the stepwise fitness gain of each additional driver mutation. These data illustrate that genetic diversity within a tumour population is not necessarily indicative of an elevated mutation rate, but instead may signify that tumour clones are experiencing only weak selection.

We note that our model makes a number of gross oversimplifications in describing carcinogenesis that could potentially impact the model dynamics. First, driver genes in our model represented only proto-oncogenes or haploinsufficient tumour suppressor genes, that is, a cell acquiring a mutation to a single driver gene locus gained an increase in fitness. In reality, many driver genes are recessive tumour suppressor genes, and so no increase in fitness is caused until both a selectively neutral first hit, and a second hit are acquired at the tumour suppressor locus. In this regard, a mutator phenotype might cause particularly efficient tumorigenesis if many selectively neutral first hits are required for cancer production. Second, we assumed that mutations accrue in a monotonic stepwise fashion. The mutator phenotype CIN is a hallmark of many cancer types (Rajagopalan et al. 2003): CIN tumours frequently show whole- or part-chromosome arm aberrations, wherein many genes located on a particular chromosome have been ‘mutated’ in a single mutational event. Also, copy number gains are frequently observed in CIN; genetic gains are potentially reversible events, as additional genic copies may later be lost. Thus, CIN cancers are unlikely to accrue mutations in the monotonic stepwise fashion we have described in our model, and so, the dynamics of the evolution of CIN may differ from our model's predictions. Third, we have assumed that mutator genes are themselves selectively neutral, that is, that mutations of mutator genes do not cause an increase in fitness. Some driver genes may double as mutator genes; for example, mutation of the intestinal tumour suppressor gene *APC* is sufficient for tumorigenesis (Lamlum et al. 2000), but may also instigate CIN (Fodde et al. 2001); similarly mutations to the *TP53* tumour suppressor are usually considered driver mutations and are associated with genetic instability (Song et al. 2007). Selection dominates evolution in our model, and so direct selection for mutator genes would likely alter the dynamics. Fourth, we have assumed that all driver genes have equivalent effects on fitness when mutated and, similarly, that all genes are equally likely to be mutated. The patterns of mutation and selection would likely alter if this unrealistic assumption were relaxed. Fifth, we have neglected to describe spatial heterogeneity within a tumour. Clonal interference, whereby two spatially adjacent clones with similar fitness impede each other's growth, slows the rate of tumour evolution (Martens et al. 2011). Such spatial considerations may also affect the evolution of mutator clones. Relatedly, we have assumed that the fitness of cells within the tumour is strictly relative; this leads to the least fit cells being rapidly outcompeted by fitter cells, and so clones carrying deleterious housekeeper mutations are rapidly driven to extinction. Relaxing this assumption, for instance by competing cells only with their ‘neighbours’, may alter the clonal dynamics (and indeed overall tumour dynamics), particularly for clones bearing deleterious mutations.

In summary, we have constructed a model of the evolution of genetic instability during tumour progression. Our model incorporates clonal expansions and deleterious mutations and allows the mutation rate to evolve, whereas previous models have considered only some of these issues. Our model predicts that the strength of selection for additional driver mutations determines whether or not a cancer is likely to evolve a mutator phenotype and suggests that future efforts should be devoted to measuring the degree of fitness effects of mutations in carcinogenesis.

## Supporting Information

**Figure S1.** Housekeeper mutations and an evolving mutation rate with moderate to strong selection.

**Figure S2.** Requiring different numbers of driver mutations for cancer.

**Figure S3.** Abundant deleterious mutations and the evolution of an increased mutation rate.
