# Supplementary figures and images for: Modelling the evolution of genetic instability during tumour progression
Source: Evol Appl. 2012 Nov 26;6(1):20–33. doi: 10.1111/eva.12024 (PMC3567468; doi:10.1111/eva.12024)

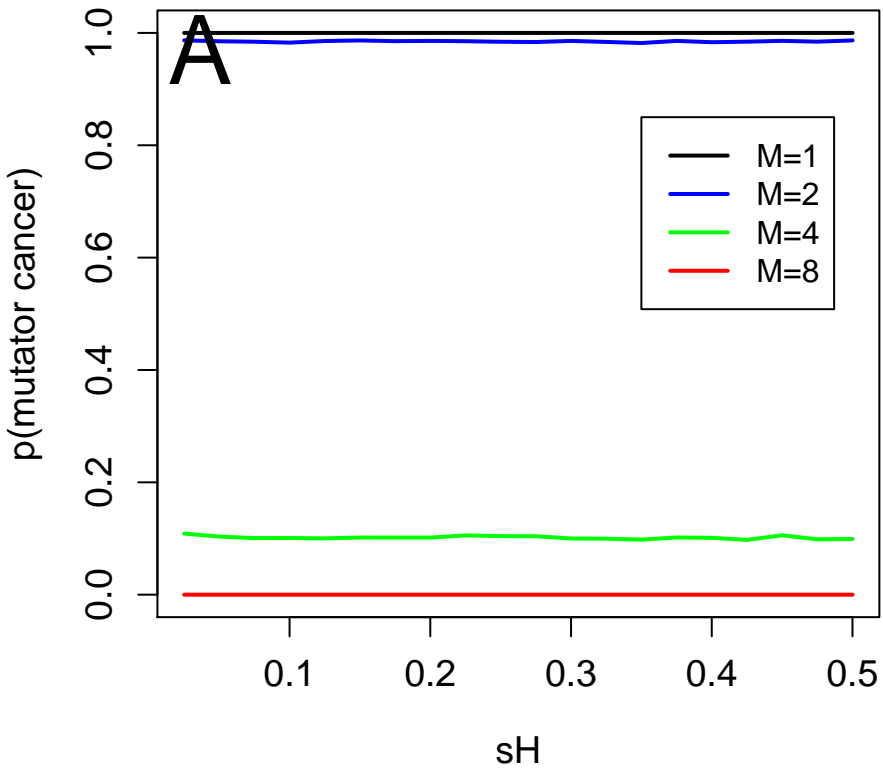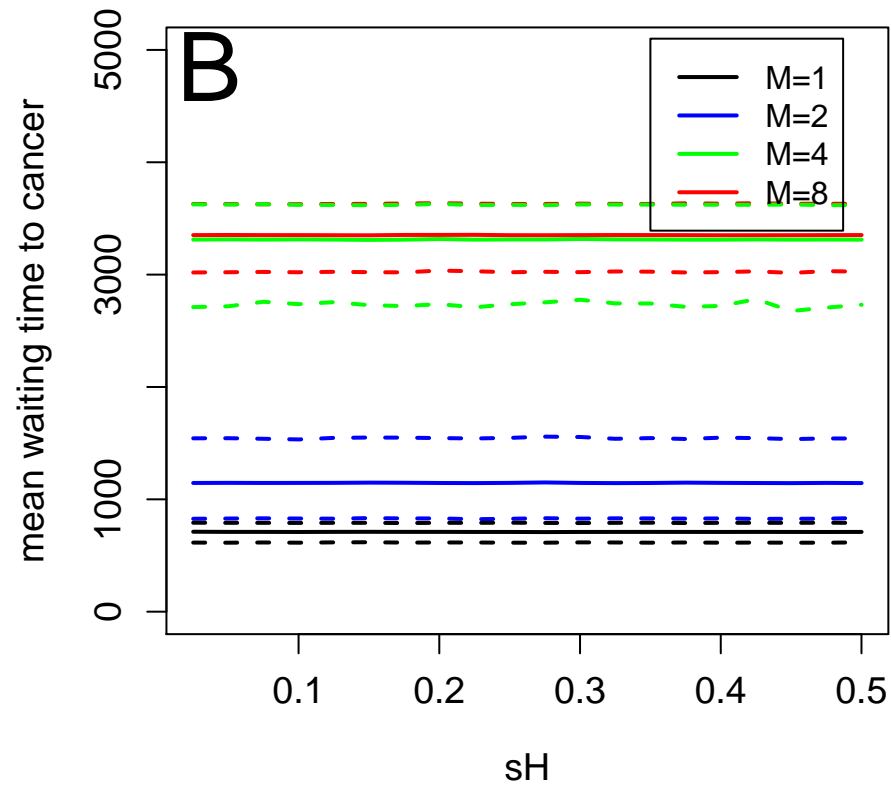

Supplement: Supplementary file 1 [file eva0006-0020-SD1.pdf]

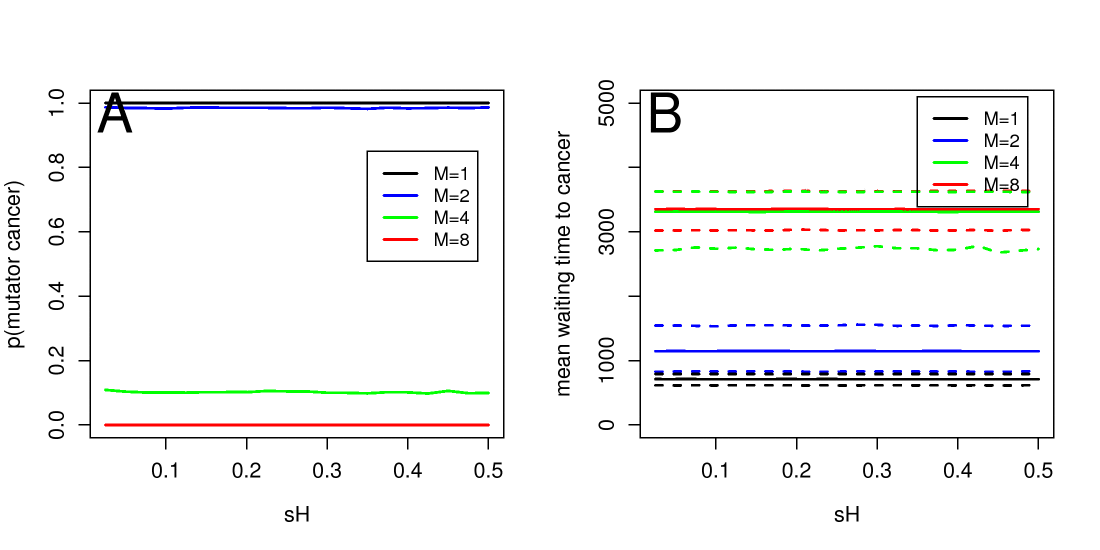

Supplement: Supplementary file 2 [file eva0006-0020-SD4.png]

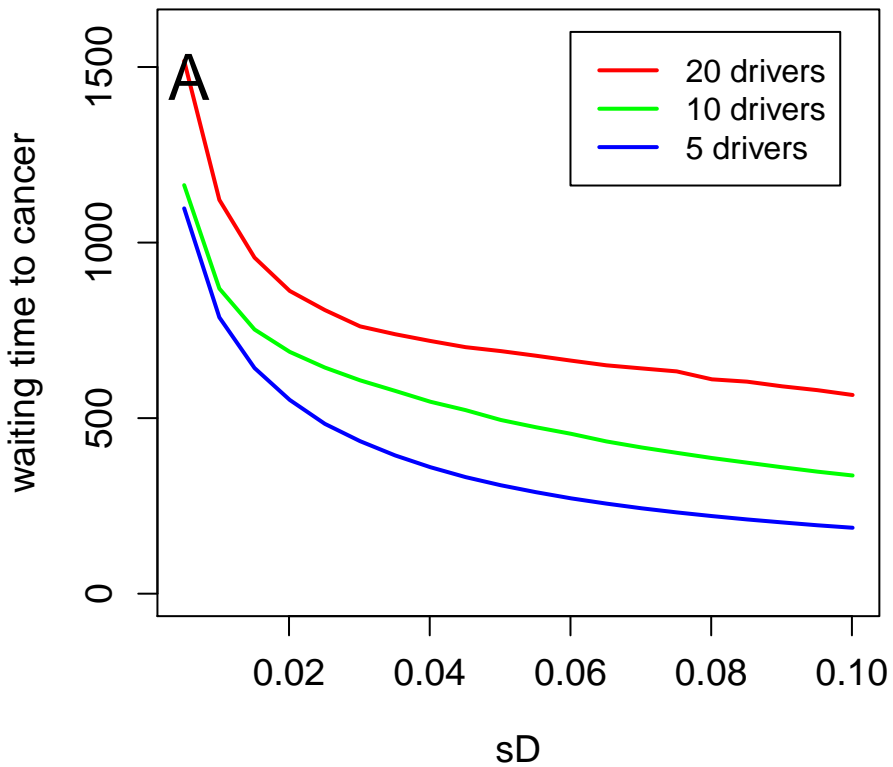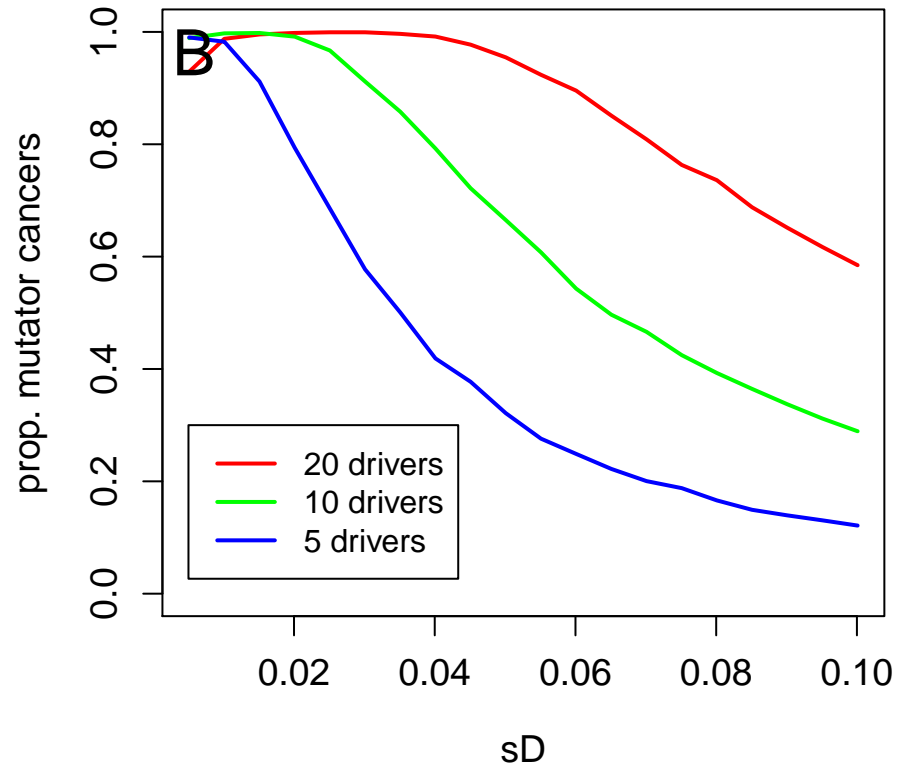

Supplement: Supplementary file 4 [file eva0006-0020-SD2.pdf]

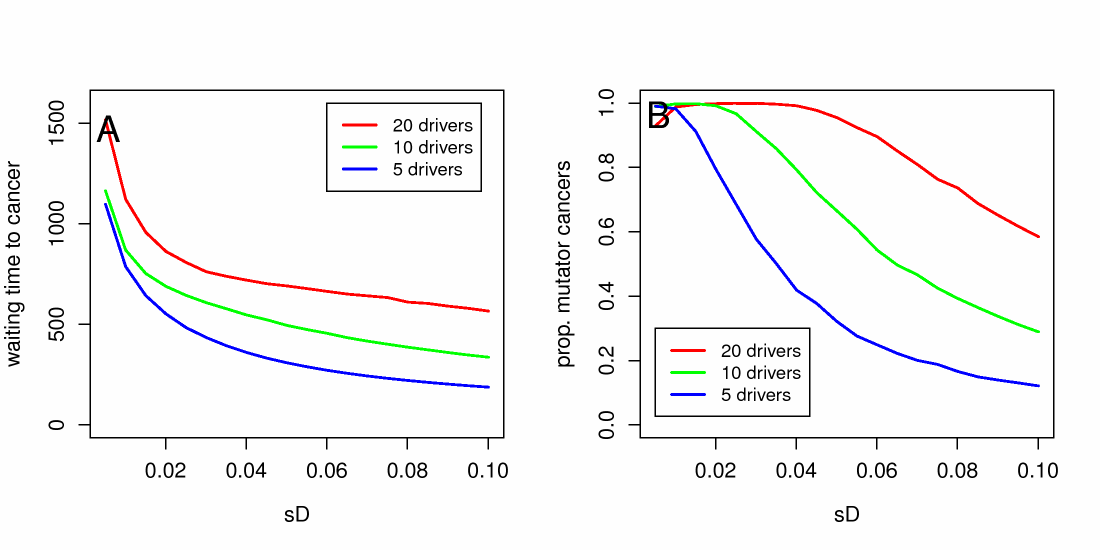

Supplement: Supplementary file 5 [file eva0006-0020-SD5.png]

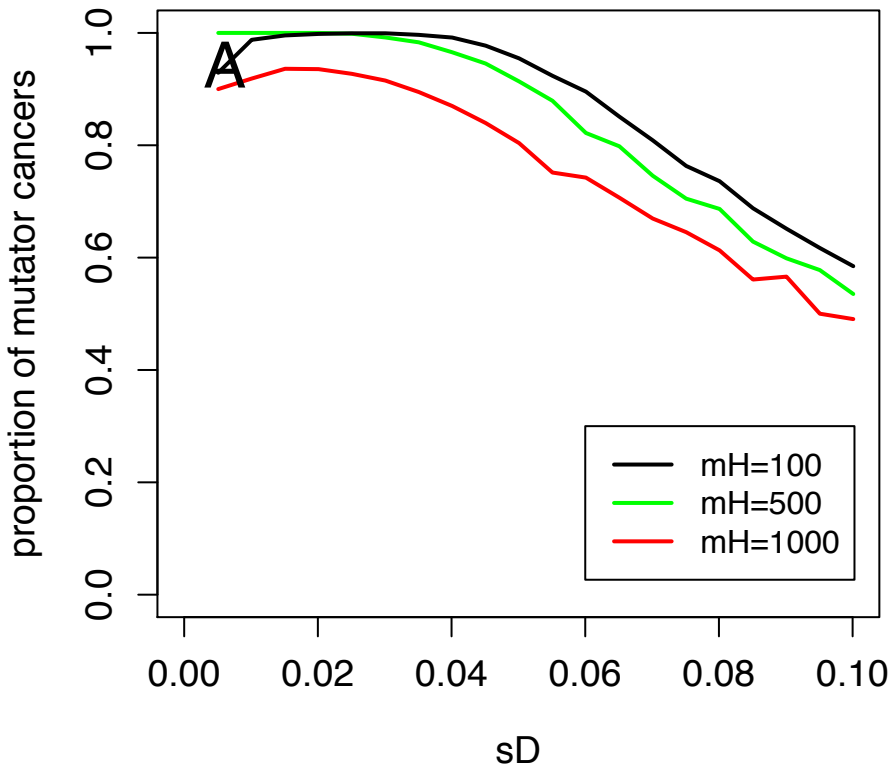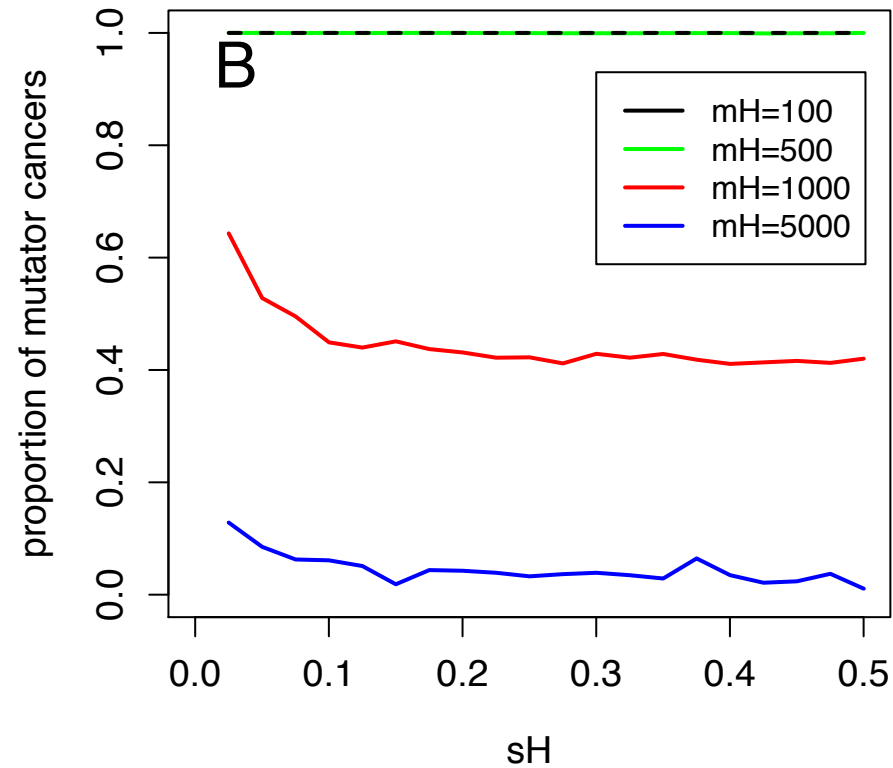

Supplement: Supplementary file 7 [file eva0006-0020-SD3.pdf]

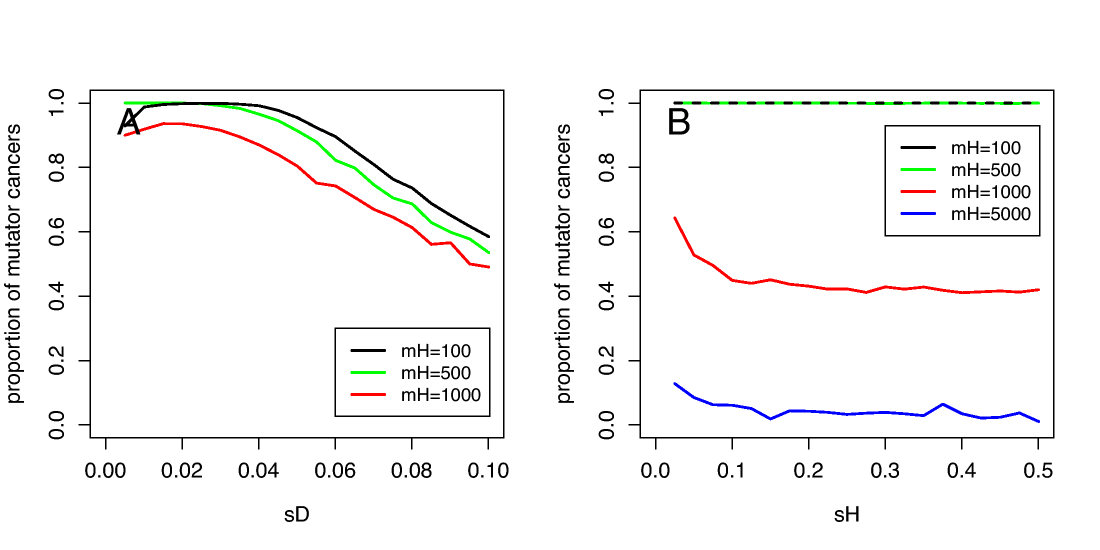

Supplement: Supplementary file 8 [file eva0006-0020-SD6.png]
